# NT-proBNP in Acute De Novo Heart Failure: A Key Biomarker for Predicting Myocardial Recovery—COMFE Registry

**DOI:** 10.3390/life15040526

**Published:** 2025-03-23

**Authors:** Raquel López-Vilella, Inés Gómez-Otero, Víctor Donoso Trenado, David García-Vega, Óscar Otero-García, Luis Martínez Dolz, José Ramón González-Juanatey, Luis Almenar Bonet

**Affiliations:** 1 Cardiology Department, Hospital Universitari i Politècnic La Fe, 46026 Valencia, Spain; vdonoso@outlook.com (V.D.T.); lualmenar@gmail.com (L.A.B.); 2 Heart Failure and Transplant Unit, Hospital Universitari i Politècnic La Fe, 46026 Valencia, Spain; martinez_luidol@gva.es; 3 Cardiology Department, Complejo Hospitalario Universitario de Santiago de Compostela, 15706 Santiago de Compostela, Spain; maria.ines.gomez.otero@sergas.es (I.G.-O.); david.garcia.vega@sergas.es (D.G.-V.); oscaroterogarci@gmail.com (Ó.O.-G.); jose.ramon.gonzalez.juanatey@sergas.es (J.R.G.-J.); 4 Instituto de Investigación Sanitaria de Santiago de Compostela (IDIS), 15706 Santiago de Compostela, Spain; 5 Centro de Investigación Biomédica en Red de Enfermedades Cardiovasculares (CIBERCV), Instituto de Salud Carlos III, 28029 Madrid, Spain

**Keywords:** de novo heart failure, improved ejection fraction, NT-proBNP, myocardial recovery

## Abstract

This study aims to analyze whether NT-proBNP at admission and discharge in de novo heart failure (HF) with reduced ejection fraction (HFrEF) is associated with myocardial recovery. This is a prospective observational study in two centers. Patients admitted with de novo HFrEF between 2021 and 2023 were included. HF with improved ejection fraction (HFimpEF) was defined as an improvement of at least 10 points with an ejection fraction >40%. Of the 248 patients who were included, 63.3% met HFimpEF criteria at follow-up, with no differences in age or gender. There were no differences in NT-proBNP at admission, but there were at discharge, where its value was inversely associated with myocardial recovery (OR 0.99 for each increase in the square root of NT-proBNP, 95% CI 0.98–0.99, *p* = 0.048). An NT-proBNP > 10,000 pg/mL at discharge was independently associated with reduced ventricular recovery (OR 0.28, 95% CI 0.07–0.94, *p* = 0.043). A smaller reduction in NT-proBNP during admission decreased the probability of recovery (OR 0.13, 95% CI 0.03–0.61, *p* = 0.010). In conclusion, in admissions for de novo HFrEF, NT-proBNP at discharge is inversely associated with myocardial recovery; a level > 10,000 pg/mL is an independent predictor for a lack of recovery, while a greater reduction increases the likelihood of recovery.

## 1. Introduction

Heart failure (HF) represents a significant burden on public health, with a prevalence that is steadily increasing worldwide. This chronic condition affects millions of people, with substantial morbidity and mortality, as well as a considerable impact on patients’ quality of life [1]. Despite advances in treatment, HF remains one of the leading causes of hospitalization and mortality, with a five-year mortality rate exceeding that of many common cancers [1,2]. Within HF, the onset of de novo HF with decompensation may indicate rapid disease progression and a higher risk of complications, underscoring the importance of close clinical monitoring and early therapeutic intervention [3]. Currently, the amino-terminal fragment of type B natriuretic peptide (NT-proBNP) is recognized as a crucial biomarker in the management of both chronic HF and de novo HF [4]. Its value in risk stratification, differential diagnosis, and treatment monitoring in HF patients has been widely acknowledged [4,5]. However, in HF with reduced left ventricular ejection fraction (HFrEF), it is not yet clear whether NT-proBNP levels or their evolution during a first hospitalization for HF can predict ventricular function recovery over time. HF with recovered or improved ejection fraction (HFrecEF) is defined as cases where the ejection fraction (EF) improves from ≤40% to ≥50% [1]. This subgroup of patients has gained increasing relevance over the past decade, with an estimated prevalence of 10–40% among HF patients [6]. Improvement in ejection fraction is primarily attributed to the use of disease-modifying therapies, the optimal management of comorbidities, and other factors [6,7]. Although patients with HFrecEF tend to have a better prognosis compared to those with persistent HFrEF, they remain at elevated risk of adverse cardiovascular events and hospital readmissions. Therefore, identifying these patients, ensuring continuous follow-up, and optimizing treatment are essential [6,8]. The primary objective of this study was to analyze, in patients admitted with de novo HFrEF (first episode), whether plasma NT-proBNP levels at admission and discharge are associated with myocardial recovery over time. The secondary objective was to assess whether changes in this biomarker during hospitalization (from admission to discharge) are related to subsequent myocardial recovery.

## 2. Materials and Methods

**Figure 1:**
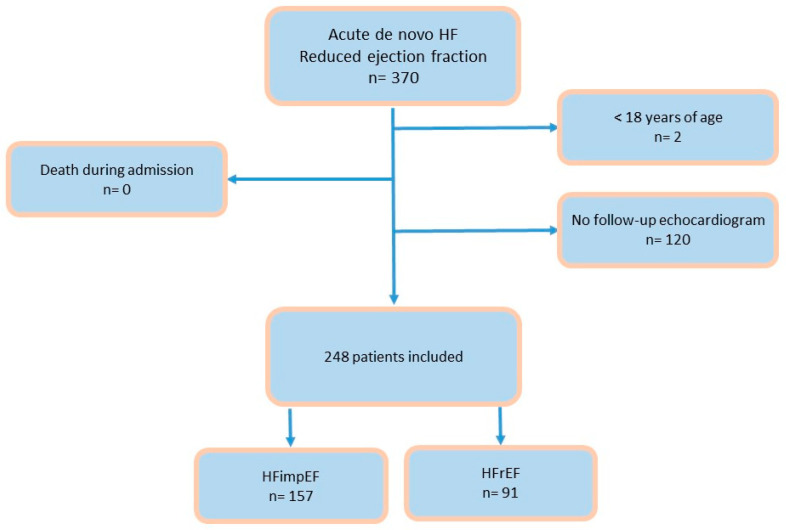
Flow chart. Abbreviations: HF: heart failure; HFimpEF: heart failure with improved ejection fraction; HFrEF: heart failure with reduced ejection fraction.

A prospective observational study was conducted at two referral centers in Spain. All consecutive patients admitted with a diagnosis of de novo HF with reduced ejection fraction (40% or less) between March 2021 and December 2023 were included. Only those patients in whom acute HF presented as the first manifestation of heart failure were considered, meaning patients who had been previously diagnosed with heart failure before the index admission were not included [1]. Exclusion criteria included patients with a prior HF diagnosis, those with an initial ejection fraction greater than 40%, pediatric patients, and those who died during hospitalization. Patients without a follow-up echocardiogram were also excluded. A total of 370 patients were recruited; two were excluded due to being under 18 years of age, and the remainder were excluded for lacking a follow-up echocardiogram. No patients died during hospitalization. Ultimately, 248 patients were included in the study (Figure 1).

This is an interim analysis of a long-term prospective open database; it did not aim to analyze the causes or mechanisms underlying the relationship between pro-BNP and acute heart failure, but rather to analyze the prognostic value of NT-proBNP at discharge, as well as its reduction during admission in acute heart failure.

Variables included clinical, echocardiographic, prognostic, therapeutic, and biomarker data. Patient demographic, clinical, laboratory, and echocardiographic characteristics were analyzed at admission and during follow-up. Laboratory variables, including NT-proBNP, were also assessed at discharge. Improved ejection fraction (HFimpEF) was defined as an improvement in ejection fraction of at least 10 percentage points compared to baseline, with a follow-up ejection fraction exceeding 40%.

The study was approved by the Biomedical Research Ethics Committee of both hospitals, and the ethical principles outlined in the Declaration of Helsinki for medical research involving human subjects were followed.

### Statistical Analysis

Initially, baseline characteristics between the groups were compared. Quantitative variables approximating a normal distribution according to the Kolmogorov–Smirnov test were expressed as means (standard deviation) and compared using Student’s *t*-test. Non-normally distributed variables were presented as the median (interquartile range) and compared using the Mann–Whitney U test. Categorical variables across groups were compared using Pearson’s chi-square test. To analyze the influence of NT-proBNP at discharge on myocardial recovery, two approaches were used. In the first, since a linear relationship between the variable of interest and NT-proBNP was not anticipated, NT-proBNP values were transformed to meet the linearity assumption, using the square root of NT-proBNP as the independent variable. Alongside NT-proBNP at discharge, additional confounding variables were included to control their influence on model variability. In the second approach, NT-proBNP at discharge was categorized into five groups, and a logistic regression model was applied, considering the same variables as in the first model. Lastly, the relationship between the variation in NT-proBNP from admission to discharge and myocardial recovery was also analyzed. The NT-proBNP discharge/admission ratio was calculated, and since this did not meet the linearity assumption, it was transformed using the cube root of the ratio.

## 3. Results

### 3.1. Baseline Characteristics

Of the 248 patients included, 157 (63.3%) met the criteria for HFimpEF in the follow-up echocardiography, while 91 patients (36.7%) continued to have reduced EF. The average duration of follow-up was 509 days (267–730). The baseline characteristics of both groups are shown in Table 1. There were no significant differences between the groups regarding age or sex. The average age was approximately 70 years, with a male-to-female ratio of 3:1. Regarding comorbidities, the group with persistent reduced EF had more cardiovascular risk factors, although without statistically significant differences except for chronic kidney disease (more frequent in the non-recovery group) and peripheral artery disease (trend toward significance). The average EF from the initial echocardiography was similar in both groups (28%). No differences were observed in NT-proBNP levels at admission between the groups that improved EF and those that did not. However, NT-proBNP at discharge was lower in patients whose cardiac function improved over time.

### 3.2. Relationship Between NT-proBNP at Discharge and Myocardial Recovery

**Figure 2:**
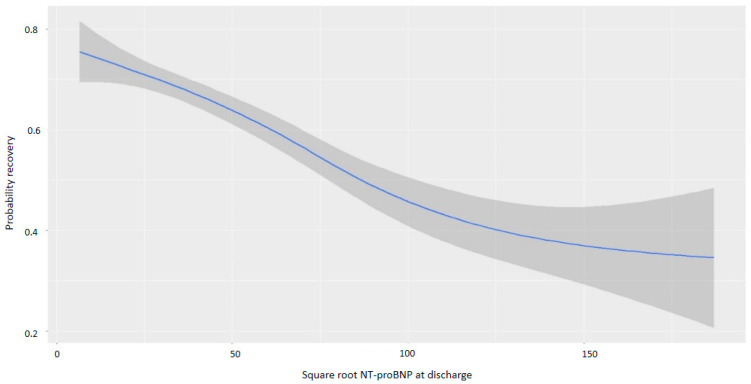
Probability of myocardial recovery based on the square root of NT-proBNP at discharge. Abbreviations: NT-proBNP: N-terminal fragment of B-type natriuretic peptide.

**Figure 3:**
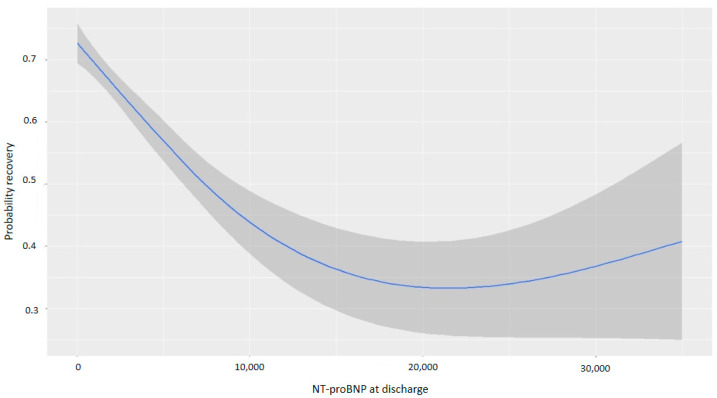
Probability of myocardial recovery based on NT-proBNP at discharge. Abbreviations: NT-proBNP: N-terminal fragment of B-type natriuretic peptide.

**Figure 4:**
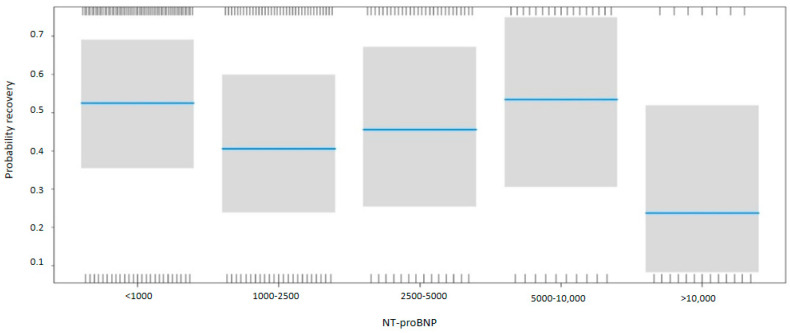
Probability of myocardial recovery based on the NT-proBNP. Multivariate analysis. Abbreviations: NT-proBNP: N-terminal fragment of B-type natriuretic peptide.

NT-proBNP levels at discharge were inversely associated with myocardial recovery. The odds ratio (OR) for myocardial recovery was 0.99 for every one-point increase in the square root of NT-proBNP, indicating that higher NT-proBNP levels at discharge were associated with a lower probability of recovery (Table 2 and Figure 2). Certain underlying HF etiologies, such as tachycardiomyopathy, idiopathic causes, and valvular disease, were also associated with recovery (Table 2). Figure 3 shows that higher NT-proBNP levels at discharge reduced the probability of recovery in absolute terms. In the multivariate analysis by categories, NT-proBNP levels above 10,000 pg/mL at discharge were independently associated with the non-recovery of ventricular function (OR 0.28, *p* = 0.043) (Table 3). Patients with NT-proBNP levels above 10,000 pg/mL had a less than 20% likelihood of meeting the criteria for improved EF (Figure 4).

### 3.3. Impact of NT-proBNP Reduction During Hospitalization

**Figure 5:**
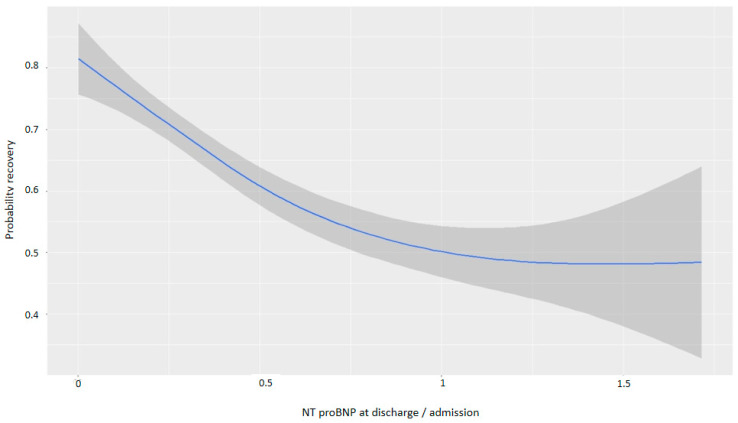
Graph showing the relationship between the reduction in NT-proBNP and the probability of recovery (discharge/admission).

**Figure 6:**
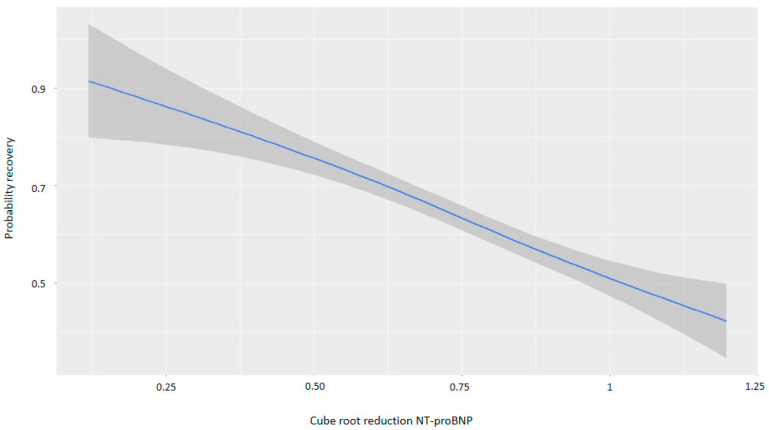
Graph showing the relationship between the cube root of the reduction in NT-proBNP and the probability of recovery. Abbreviations: NT-proBNP: N-terminal fragment of B-type natriuretic peptide.

**Figure 7:**
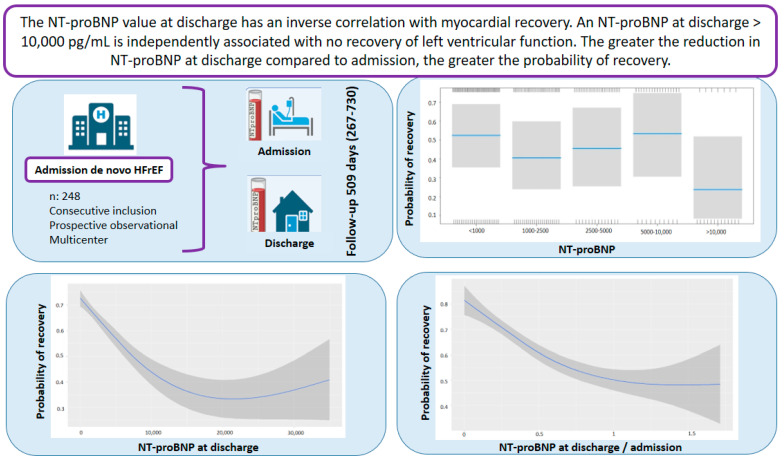
Graphical abstract. Abbreviations: HFrEF: heart failure with reduced ejection fraction; NT-proBNP: N-terminal fragment of B-type natriuretic peptide.

For every one-point increase in the square root of the NT-proBNP discharge/admission ratio, the likelihood of recovery decreased, with an OR of 0.13 (Table 4). Figure 5 and Figure 6 demonstrate that greater reductions in NT-proBNP levels from admission to discharge were associated with higher probabilities of myocardial recovery. Figure 7 presents a graphical summary of the main results of the analysis.

## 4. Discussion

Heart failure is one of the leading causes of morbidity and mortality worldwide, representing a significant challenge for healthcare systems due to its high prevalence (affecting more than 64 million people globally) and complexity in management [1,5]. In particular, HFrEF is associated with a poor prognosis, including an increased risk of hospitalization and mortality [9], even in the case of a first episode of acute heart failure (de novo HF) [3]. Thus, morbidity and mortality rates for HFrEF remain high, with a 5-year survival rate of 50% after hospitalization [9]. On the other hand, the improvement and/or recovery of left ventricular ejection fraction (LVEF) in the course of heart failure patients is not uncommon. Myocardial recovery has been attributed to factors such as younger age, female sex, the absence of comorbidities, non-ischemic etiology, and disease-modifying therapy [6,7,8]. Low levels of analytical biomarkers such as NT-proBNP have also been associated with recovery, but this has only been analyzed in chronic heart failure [10]. This natriuretic peptide is essential both in early diagnosis and in evaluating prognosis and monitoring treatment response in heart failure patients. Its measurement allows for risk stratification, the optimization of therapeutic interventions, and, ultimately, improvement in clinical outcomes [11]. However, it is not known whether, in a first admission for acute de novo heart failure in patients with reduced LVEF, the values at admission and discharge can predict the recovery of ventricular function over time. This study has shown that NT-proBNP levels at discharge from this first hospitalization are associated with the recovery of ventricular function over time. Thus, NT-proBNP levels at discharge are inversely associated with myocardial recovery, and with NT-proBNP levels greater than 10,000 pg/mL, the percentage of patients meeting the criteria for improved LVEF is less than 20%. Additionally, a reduction in NT-proBNP levels at discharge compared to admission is associated with recovery, so the greater the difference between these two values, the higher the probability of myocardial recovery.

In this analysis, the majority of patients were male, with a mean age of 69 years. This is similar to other studies on de novo heart failure. In the TIDY-HF study (which included 82.4% of patients diagnosed during a first hospitalization), the mean age was 65.5 years, also with a majority of men [12]. In the STRONG-HF clinical trial, the age was even lower, at 63 years, with 61% male participants [13]. The average LVEF was lower than in other studies of patients with de novo heart failure; however, the mean NT-proBNP level was similar, as was the profile of cardiovascular risk factors, with hypertension being the most prevalent [14].

In the follow-up, 63% of patients met the criteria for improved LVEF. Other studies have reported a wide variability in the prevalence of improved LVEF, ranging from 9% to 40%, due to different definitions used regarding the magnitude of the change in LVEF and the time elapsed between follow-up echocardiograms [15,16,17]. The group that met the criteria for improved LVEF during follow-up was slightly younger, while the group with no improvement in LVEF had more cardiovascular risk factors, particularly chronic kidney disease and peripheral artery disease (more frequent in the non-recovery group). There is a trend toward an association between hypertensive etiology and recovery, although it does not reach statistical significance; this may be due to the small number of hypertensive patients. Advanced age has been described as a negative predictor for recovery in some studies [18]. However, the most frequently described predictors of myocardial function recovery in the literature are non-ischemic cardiomyopathy, left bundle branch block, recent HF diagnosis, atrial fibrillation, and female sex [19,20].

Regarding analytical biomarkers, as they generally reflect different mechanistic pathways of myocardial injury and repair, some, such as natriuretic peptides (ventricular remodeling), troponin (myocardial injury), and ST2 (inflammation), can provide independent additive prognostic information in HFrEF [4,19,21,22]. For instance, elevated NT-proBNP levels at admission in acute heart failure are an independent predictor of prolonged hospitalization, along with renal failure [23]. In addition, in HFrEF, it is well known that a greater reduction in NT-proBNP during treatment with optimal medical therapy is associated with greater improvements in LVEF and better clinical outcomes [24]. The STRONG-HF trial showed that rapid up-titration of the recommended medical therapy in an intensive care strategy reduced clinical events, regardless of initial NT-proBNP values [13]. A pre-specified echocardiographic analysis from the GUIDE-IT trial examined the degrees of reverse remodeling associated with changes in NT-proBNP concentrations in 269 patients, finding a reduction in left ventricular volumes and an improvement in LVEF proportional to the degree of NT-proBNP reduction [23].

In this study, no significant association was found between medical treatment at discharge and the recovery of ventricular function over time. Some of these factors showed trends but did not reach statistical significance, possibly due to sample size or the distinct characteristics of de novo HF patients compared to chronic HF populations. The management of HFrEF is based on guideline-directed medical therapy (GDMT). Contemporary GDMT includes four main pharmacologic pillars: beta-blockers (BB), angiotensin receptor-neprilysin inhibitors (ARNi), mineralocorticoid receptor antagonists (MRA), and sodium-glucose cotransporter 2 inhibitors (SGLT2i). The aggregated effect of GDMT has been explored in several meta-analyses [25]. Vaduganathan et al. [7] compared the new quadruple therapy (ARNi + BB + MRA + SGLT2i) to previous angiotensin-converting enzyme inhibitors and/or angiotensin receptor blockers (ACEi and/or ARB) + BB, observing a 47% reduction in all-cause mortality. This was accompanied by an increase of 1.4 to 6.3 years of survival compared to conventional therapy (ACEi and/or ARB + BB). Two additional analyses were performed before the introduction of SGLT2 inhibitors. The combination of ARNi + BB + MRA showed a reduction in all-cause mortality of approximately 62%. However, it is important to consider that most studies have used populations treated with heterogeneous GDMT regimens. Therefore, it is difficult to apply prevalence estimates using diverse treatment regimens to all patients with systolic dysfunction.

Regarding the impact of NT-proBNP reduction during hospitalization for acute HF, when analyzing the relationship between this biomarker and clinical events, the earliest studies by Bettencourt et al. indicated that those patients who experienced more clinical events at 6 months had a smaller decrease in BNP levels from hospital admission to discharge [26]. A 2022 sub-analysis from the CLUSTER-HF study analyzed NT-proBNP levels at discharge in a subgroup of 94 patients, finding that a smaller absolute decrease in NT-proBNP (less than 3350 pg/mL) at discharge from hospitalization for heart failure was associated with fewer clinical events at 180 days (emergency visits, rehospitalization for heart failure, and death from any cause); however, this sub-analysis did not examine the relationship between NT-proBNP at discharge and ventricular function at follow-up [27].

It is important to consider that until now, the relationship between NT-proBNP at discharge after a first admission and the evolution of this biomarker during the first hospitalization with the recovery of ventricular function at follow-up had not been analyzed.

It is possible that a greater reduction in NT-proBNP during hospitalization and NT-proBNP levels at discharge could serve as an early sign of good evolution. It is also possible that a low value and a more significant reduction during hospitalization are associated with more intensive treatment during the hospitalization, although it is not common nor is it protocolized for treatment in acute HF admissions to be guided by natriuretic peptide levels. What is relevant is that there is growing interest in understanding the natural history and prognosis of patients who experience an improvement in LVEF under treatment. Understanding the information provided by this study helps clinicians identify patients who are less likely to experience myocardial recovery and in whom treatment and follow-up for clinical assessment and device indication should be particularly thorough.

The most important limitation of this study is inherent to patient registry databases, as it is an observational study. The observational nature of the study presents a significant limitation, which is the considerable number of patients excluded for various reasons. The main reason for exclusion was the lack of follow-up echocardiograms. This occurred because, as a referral hospital, some patients continue their outpatient follow-up in their corresponding healthcare area after discharge. On the other hand, the etiology is heterogeneous. Idiopathic etiology was established after ruling out valvular diseases, arrhythmias, and coronary artery disease through coronary angiography or stress cardiac magnetic resonance imaging. However, no information has been collected regarding the treatment of ischemic cardiomyopathy or valvular disease, for instance. In addition, it should be considered that this is primarily a descriptive study that aimed to point out a novel clinical revelation of great interest. However, at this stage, we have not delved into the molecular mechanisms that condition myocardial reversibility after cardiac injury or the molecular role of NT-proBNP in this process. It will be of interest in the near future to use experimental in vitro or in vivo models to further explore these results. Finally, the sample size may limit the statistical power of the findings. However, it presents a fairly precise methodology, as the data were prospectively entered as patients were admitted and by the same individual in each center, which ensures reliability and prevents biases among individuals. Furthermore, the recruitment period was 3 years, a sufficient timeframe to obtain a significant number of patients. It is important to note that this study is one of the first to specifically evaluate the evolution of NT-proBNP in patients with de novo HFrEF and its relationship with myocardial recovery. In contrast to previous studies focusing on chronic heart failure or exacerbations, our findings provide evidence for the prognostic relevance of NT-proBNP in an as-yet underexplored setting. This information reinforces the value of NT-proBNP not only as a diagnostic marker but also as a prognostic tool in the management of de novo heart failure. These findings may help clinicians to identify patients at higher risk of persistent dysfunction, allowing for more intensive treatment titration and closer monitoring after hospital discharge.

## 5. Conclusions

In patients with a first admission for de novo heart failure with reduced left ventricular ejection fraction, NT-proBNP levels at discharge are inversely associated with myocardial recovery. Specifically, NT-proBNP levels at discharge greater than 10,000 pg/mL are independently associated with the lack of recovery of left ventricular function. The greater the reduction in NT-proBNP concentrations at discharge compared to admission, the higher the probability of myocardial recovery during follow-up. As an observational study, this represents an initial step toward understanding NT-proBNP in de novo heart failure, highlighting the need for future experimental validation.

## Figures and Tables

**Table 1:** Baseline characteristics of the study population.

Variables	HFimpEF YES n: 157	HFimpEF NO n: 91	*p*
Age (years)	69 (58–76)	71 (61–78)	0.353
Male (n,%)	120 (76.43)	68 (74.73)	0.882
Hypertension (n,%)	92 (58.60)	63 (69.23)	0.126
Dyslipidemia (n,%)	73 (46.50)	48 (52.74)	0.414
Diabetes (n,%)	43 (27.39)	34 (37.36)	0.135
CKD (n,%)	28 (17.83)	29 (31.87)	0.018
Stroke (n,%)	8 (5.12)	8 (8.79%)	0.390
CPOD (n,%)	11 (7.00)	10 (10.99)	0.396
SAHS (n,%)	7 (4.49)	7 (7.69)	0.444
PAD (n,%)	9 (5.7)	12 (13.33)	0.068
Alcohol (n,%)	44 (28.03)	21 (23.08)	0.481
Smoking (n/%)	64 (40.76)	26 (28.57)	0.074
Time to titration/optimization (days)	117 (38.25–186.25)	110 (44.50–172.00)	0.837
Etiology of HF (n, %)			
Hypertension	10 (6.37)	3 (3.30)	0.453
Tachycardiomyopathy	46 (29.30)	12 (13.19)	0.006
Ischemic	27 (17.20)	40 (43.96)	<0.0001
Idiopathic	32 (20.38)	20 (21.98)	0.892
Valvular	12 (7.64)	1 (1.10)	0.050
Other	26 (19.11)	15 (16.48)	0.729
Creatinine at discharge (mg/dL)	1.10 (0.89–1.37)	1.14 (0.87–1.51)	0.361
GFR at discharge (mL/min/1.73 m^2^)	65.93 (21.63)	62.00 (24.44)	0.217
NT-proBNP at admission (ng/mL)	4695 (2280–8623.0)	5069 (2166–11,324.5)	0.439
NT-proBNP at discharge (ng/mL)	1464 (607.5–3316.5)	2349 (917.0–5943.0)	0.006
LVEF at admission	28.90 (6.44)	28.78 (7.38)	0.90
LVEF after titration	51.58 (7.249)	32.916 (6.60)	<0.0001
SBP at admission (mmHg)	149 ± 24	154 ± 14	0.067
DBP at admission (mmHg)	76 ± 11	78 ± 13	0.195
SBP at discharge (mmHg)	121 ± 15	124 ± 17	0.458
DBP at discharge (mmHg)	65 ± 12	66 ± 13	0.452
4 Pillars at discharge (n, %)	103 (66.03)	53 (58.24)	0.277
4 Pillars after optimization (n, %)	110 (72.37)	57 (64.04)	0.227
RAASi at discharge (n, %)	142 (90.45)	76 (83.52)	0.158
Beta-blockers at discharge (n, %)	142 (91.03)	88 (96.70)	0.150
Mineralocorticoid receptor antagonists at discharge (n, %)	130 (82.80)	69 (75.82)	0.244
SGLT2i at discharge (n, %)	131 (83.39)	76 (83.52)	0.126
RAASi after optimization (n, %)	139 (90.26)	74 (81.32)	0.070
Beta-blockers after optimization (n, %)	147 (93.63)	88 (97.78)	0.250
Mineralocorticoid receptor antagonists after optimization (n, %)	128 (82.58)	74 (81.32)	0.939
SGLT2i after optimization (n, %)	144 (91.72)	80 (88.89)	0.611
Duration of admission in days	7.00 (5.00–11.00)	7.00 (5.00–10.50)	0.636
Weight at discharge (kg)	72.00 (61.85–86.00)	74.50 (63.62–90.00)	0.547
NYHA first consultation (I-IV)	2.00 (1.00–2.00)	2.00 (2.00–2.00)	0.782
Time to titration (days)	115.00 (38.5–185.5)	110.0 (44.5–166.0)	<0.001

Abbreviations: DBP: diastolic blood pressure; COPD: chronic obstructive pulmonary disease; CKD: chronic kidney disease; GFR: glomerular filtration rate; HFimpEF: heart failure with improved ejection fraction; LVEF: left ventricular ejection fraction; NT-proBNP: N-terminal pro-B-type natriuretic peptide; NYHA: New York Heart Association; PAD: peripheral artery disease; RAASi; renin–angiotensin–aldosterone system inhibitors; SAHS: sleep apnea/hypopnea syndrome; SBP: systolic blood pressure; SGLT2: sodium-glucose cotransporter-2 inhibitors.

**Table 2:** Logistic model for NT-proBNP at discharge.

Variable	OR	Low	High	*p*-Value
√NT-proBNP at discharge	0.990	0.980	0.999	0.048
√Age	0.923	0.646	1.300	0.652
Sex (female)	0.911	0.458	1.835	0.792
Peripheral artery disease	0.484	0.167	1.366	0.171
Hypertensive etiology	3.888	1.043	18.941	0.058
Tachycardiomyopathy	4.256	1.893	10.051	<0.001
Idiopathic etiology	2.325	1.025	5.428	0.046
Valvular etiology	24.399	4.074	475.593	0.004
Other etiology	2.064	0.910	4.796	0.086
Titrated medical therapy	1.153	0.594	2.218	0.670

√ Square root. Abbreviations: NT-proBNP: N-terminal pro-B-type natriuretic peptide.

**Table 3:** Model with NT-proBNP divided into categories. Odds ratios.

	OR	Low	High	*p*-Value
(Intercept)	1.618	0.285	9515	0.588
NT-proBNP at discharge (1000–2500)	0.617	0.289	1.317	0.211
NT-proBNP at discharge (2500–5000)	0.755	0.327	1.773	0.512
NT-proBNP at discharge (5000–10,000)	1.038	0.378	2.949	0.942
NT-proBNP at discharge (>10,000)	0.28	0.077	0.939	0.043
Age	0.992	0.969	1.015	0.486
Sex (female)	0.86	0.431	1.737	0.670
Peripheral artery disease	0.52	0.174	1.532	0.233
Hypertensive etiology	4.829	1.245	24.46	0.033
Tachycardiomyopathy	4.601	2.023	11.018	<0.001
Idiopathic etiology	2.423	1.054	5.756	0.040
Valvular etiology	25.709	4.299	499.231	0.003
Other etiology	2.353	1.018	5.606	0.048
Titrated medical therapy	1.204	0.617	2.33	0.583

Abbreviations: NT-proBNP: N-terminal fragment of B-type natriuretic peptide.

**Table 4:** Logistic model for the reduction in NT-proBNP.

Variable	OR	Low	High	*p*-Value
√ Reduction in NT-proBNP	0.133	0.027	0.611	0.010
Age	0.990	0.967	1.013	0.400
Sex (female)	0.879	0.440	1.775	0.716
Peripheral artery disease	0.387	0.127	1.110	0.083
Hypertensive etiology	3.884	1.020	19.228	0.0621
Tachycardiomyopathy	4.398	1.951	10.429	<0.001
Idiopathic etiology	2.375	1.038	5.597	0.043
Valvular etiology	24.415	4.050	478.916	0.004
Other etiology	1.924	0.838	4.520	0.126
Titrated medical therapy	1.121	0.577	2.160	0.733

√ Square root. Abbreviations: NT-proBNP: N-terminal fragment of B-type natriuretic peptide.

## Data Availability

Dataset available on request from the authors.
